# Dual recombinase action in the normal and neoplastic mammary gland epithelium

**DOI:** 10.1038/s41598-021-00231-8

**Published:** 2021-10-21

**Authors:** Patrick D. Rädler, Kerry Vistisen, Aleata A. Triplett, Rayane Dennaoui, Yong Li, Hridaya Shrestha, Rosa-Maria Ferraiuolo, Amalraj Thangasamy, Dieter Saur, Kay-Uwe Wagner

**Affiliations:** 1 Department of Oncology, Wayne State University School of Medicine and Tumor Biology Program, Barbara Ann Karmanos Cancer Institute, 4100 John R, mail code EL01TM, Detroit, MI 48201 USA; 2 Eppley Institute for Research in Cancer and Allied Diseases, University of Nebraska Medical Center, 985950 Nebraska Medical Center, Omaha, NE 68198 USA; 3 Department of Internal Medicine II, Klinikum rechts der Isar, Technische Universität München, Munich, Germany

**Keywords:** Animal disease models, Cancer models, Genetic models, Disease model

## Abstract

We developed a transgenic mouse line that expresses the codon-optimized Flp recombinase under the control of the MMTV promoter in luminal epithelial cells of the mammary gland. In this report, we demonstrate the versatile applicability of the new MMTV-Flp strain to manipulate genes in a temporally and spatially controlled manner in the normal mammary gland, in luminal-type mammary tumors that overexpress ERBB2, and in a new KRAS-associated mammary cancer model. Although the MMTV-Flp is expressed in a mosaic pattern in the luminal epithelium, the Flp-mediated activation of a mutant *Kras*^*G12D*^ allele resulted in basal-like mammary tumors that progressively acquired mesenchymal features. Besides its applicability as a tool for gene activation and cell lineage tracing to validate the cellular origin of primary and metastatic tumor cells, we employed the MMTV-Flp transgene together with the tamoxifen-inducible Cre recombinase to demonstrate that the combinatorial action of both recombinases can be used to delete or to activate genes in established tumors. In a proof-of-principle experiment, we conditionally deleted the JAK1 tyrosine kinase in KRAS-transformed mammary cancer cells using the dual recombinase approach and found that lack of JAK1 was sufficient to block the constitutive activation of STAT3. The collective results from the various lines of investigation showed that it is, in principle, feasible to manipulate genes in a ligand-controlled manner in neoplastic mammary epithelial cells, even when cancer cells acquire a state of cellular plasticity that may no longer support the expression of the MMTV-Flp transgene.

## Introduction

Over the past two decades, genetically engineered mouse models have been instrumental in unraveling the biological significance of genes within signaling networks that control the development of the mammary gland as well as their roles for the onset and progression of breast cancer^1,2^. An important milestone in the development of genetically defined models was the adaptation of the bacteriophage Cre/lox recombination system for the use in mouse genetics and its subsequent application to manipulate genes in specific cell types, such as the mammary epithelium^3–7^.

A major rationale for developing Cre/lox-based conditional knockout models was to circumvent the early embryonic lethality of conventional knockouts, particularly for those genes that play key roles in breast cancers such as BRCA1, BRCA2, and PTEN^8–13^. The availability of different mouse strains that express Cre recombinase under the control of promoters that are active at particular stages of epithelial differentiation made it possible to investigate the function of genes within defined developmental windows during mammogenesis^14,15^. A transient expression of Cre recombinase leaves a permanent imprint on its recognition sequences in the genome of a cell and all its descendants during cell lineage diversification. This unique feature of the Cre/lox system is being used to genetically label epithelial cell lineages during normal mammary gland development^16–19^, and it is being applied to identify the cellular origin of mammary tumors in diverse cancer models^20–22^. By deleting genes in normal epithelial cells prior to tumor onset or in fully transformed cancer cells, the Cre/lox system is also employed to discriminate the function of genes and molecular pathways in mammary tumor initiation, cancer maintenance, and metastatic progression^23–25^. This genetic tool makes it possible to predict the biological relevance of candidate target genes for breast cancer prevention and treatment*.*

To understand the genetic cues that drive normal epithelial cell differentiation or the etiology of breast cancer, it is necessary to accurately model consecutive genetic events in the order they may occur in vivo. It is, however, challenging to develop animal models that permit a genetic modification of two or more endogenous loci at different stages of the developmental process or during neoplastic progression. A suitable approach is the use of two recombination systems where each recombinase recognizes its unique target sequences that are strategically placed within the mouse genome^26^. In this work, we report the generation of a novel transgenic strain that expresses the codon-optimized Flp recombinase under the control of the mouse mammary tumor virus long terminal repeat (MMTV-Flp) and how it can be applied in combination with Cre recombinase to delete and activate genes in the desired order in normal and neoplastic epithelial cells of the mammary gland.

## Results

### Generation of MMTV-Flp transgenic mice

**Figure 1:**
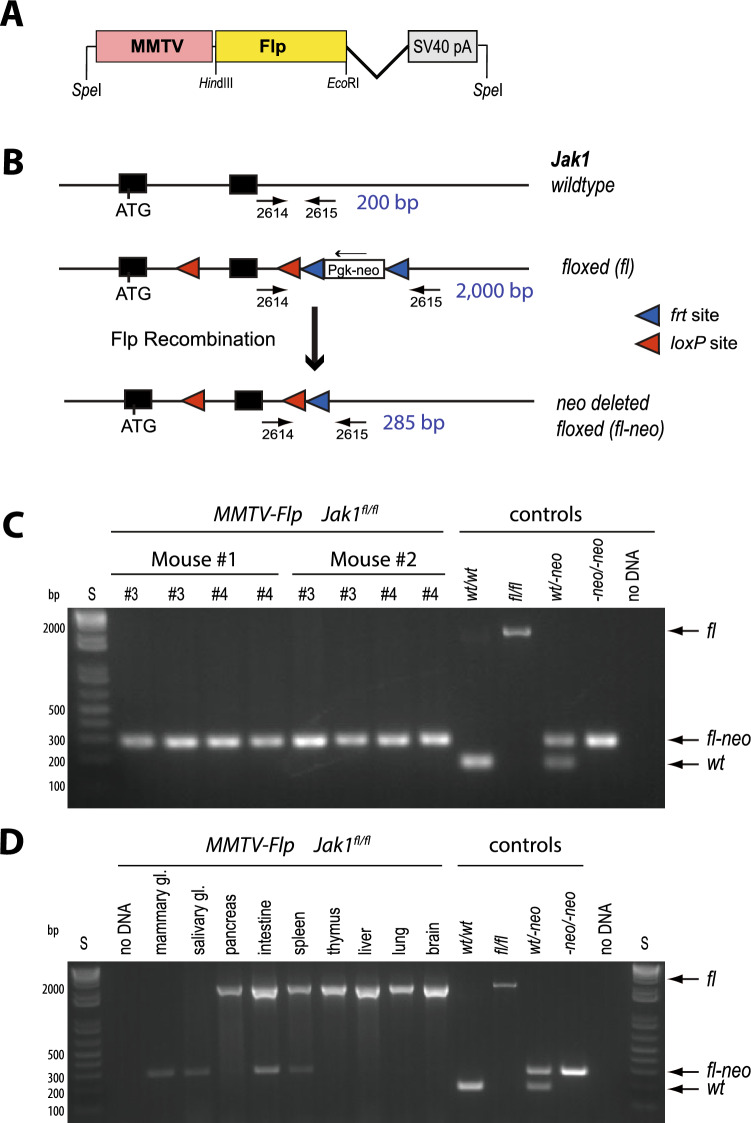
Generation of a transgenic mouse line expressing the Flp recombinase in the mammary gland. (**A**) Schematic outline of the MMTV-Flp transgene. (**B**) Diagram of the *Jak1* conditional knockout allele and its use as a reporter for the Flp-mediated excision of the neomycin (neo) selection cassette that is flanked by recognition sites (*frt*) for the recombinase. Arrows indicate the location of PCR primers to assess the Flp-mediated recombination. (**C**) PCR-based validation of the Flp-mediated recombination in the thoracic (#3) and inguinal (#4) mammary glands of two lactating MMTV-Flp *Jak1*^*fl/fl*^ females. DNA from mouse embryonic fibroblasts with defined genetic alterations in the conditional *Jak1* allele were used as controls. (**D**) PCR assay to detect Flp recombinase activity in selective organs. *MMTV* mouse mammary tumor virus long terminal repeat, *SV40 pA* intron and polyadenylation sequence (pA) of the simian polyomavirus 40, *frt* Flp recognition target site, *loxP* Cre recombinase target site, *Pgk-neo* neomycin selection marker driven by the phosphoglycerate kinase 1 (Pgk) promoter, *ATG* start codon, *S* DNA standard ladder, *Jak1* allele designation, *wt* wildtype, *fl* conditional knockout, flanked by *lox*P sites, *fl-neo fl* allele with a Flp recombinase-mediated deletion of the neomycin selection marker.

While comparing the spatial expression profiles of different MMTV-based transgenic lines^27^, we observed that the extended gene regulatory elements of the MMTV-SV40-BSSK vector facilitated a more restricted expression of exogenous proteins to the mammary gland epithelium. Therefore, we selected the extended MMTV promoter along with the SV40 intron and polyadenylation sequence to clone the MMTV-Flp transgene (Fig. 1A). The pronuclear injection of the MMTV-Flp transgene into FVB zygotes yielded four transgenic founders that were crossed with wildtype FVB/N mice to establish individual lines. Three of the four founder animals transmitted the MMTV-Flp construct to their offspring. To assess the functionality of the MMTV-Flp, we utilized a conditional *Jak1* knockout allele (*Jak1*^*fl*^) as a reporter that had been carried in the FVB/N strain^28^. An optimal expression of transgenes under the control of the extended MMTV promoter is dependent on the FVB/N strain background^2^, and the *Jak1*^*fl*^ allele was the only suitable Flp reporter strain in our colony at that time. The recombinase-mediated excision of the Pgk-neomycin selection marker from the *Jak1*^*fl*^ allele, which is surrounded by Flp recognition target (*frt*) sites, was determined by PCR. The location of the PCR primers (2614/2615) within intronic sequences downstream of the second *Jak1* coding exon is illustrated in Fig. 1B. It should be noted that in *Jak1*^*fl/wt*^ females from intermediary crosses with the MMTV-Flp, the PCR assay resulted in the co-amplification of a smaller fragment from the *Jak1* wildtype allele (*Jak1*^*wt*^) that frequently obscured the specific PCR band that resulted from the Flp-mediated excision of the Pgk-neomycin cassette (*Jak1*^*fl-neo*^). Therefore, we analyzed female mice from the three MMTV-Flp founder lines that carried the transgene in combination with two targeted *Jak1* alleles (MMTV-Flp *Jak1*^*fl/fl*^) (Fig. 1C). Using this strategy, we determined that one of three founder lines exhibited a highly consistent activation of the MMTV-Flp construct in thoracic and inguinal mammary glands of lactating mice (Fig. 1C). Moreover, the expression and functionality of the MMTV-Flp in this line were largely restricted to the mammary and salivary glands. Except for the intestine and spleen that seemed to contain cells with a recombined Pgk-neomycin cassette, none of the other organs exhibited any detectable activity of the Flp recombinase (Fig. 1D). For clarification, the PCR assay to simultaneously detect the Flp-recombined (*Jak1*^*fl-neo*^) and unrecombined (*Jak1*^*fl*^) alleles of *Jak1* is a qualitative test and may not accurately reflect the relative number of MMTV-Flp expressing cells since the amplification of the much smaller PCR fragment can outcompete the synthesis of the larger unrecombined sequence. The founder line 29542 was registered at the Mouse Genome Informatics (MGI) database as Tg(MMTV-Flp)29542Kuw (MGI:6392338) and used in all subsequent biological studies. A description of the generation of mouse embryonic fibroblasts that carry the defined *Jak1* alleles shown in Fig. 1C,D as PCR controls can be found in the “Methods” section.

### The MMTV-Flp transgene is expressed in luminal epithelial cells of the mammary gland

**Figure 2:**
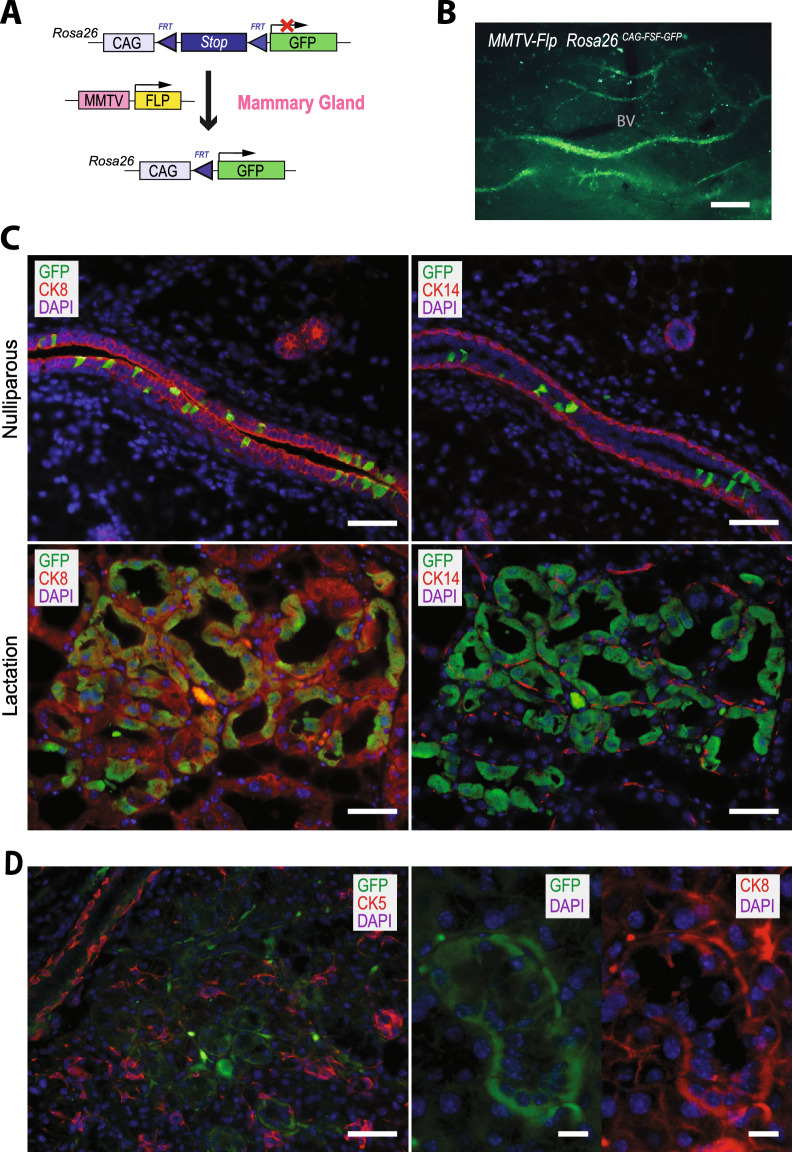
MMTV-Flp-induced expression of a GFP reporter allele in the luminal epithelium of the mammary gland. (**A**) Graphic illustration of the MMTV-Flp-mediated activation of the *Rosa26*^*CAG-FSF-CreERT2*^ reporter allele in the mammary glands of double transgenic females. (**B**) GFP-fluorescent image of mammary gland ducts of a nulliparous MMTV-Flp *Rosa26*^*CAG-FSF-CreERT2*^ double transgenic female; BV, blood vessel; bar, 1 mm. (**C**) Immunofluorescent (IF) staining of luminal cytokeratin 8 (CK8) or basal cytokeratin 14 (CK14) (both red) and GFP (green) on mammary gland sections of nulliparous (upper) and lactating (lower) double transgenic females; bars, 50 µm. (**D**) IF staining of GFP (green) in CK5 and CK8-positive epithelial cells (red) of the salivary gland; bars, 50 µm (left) and 10 µm (right).

To assess the cell-type-specific expression pattern of the MMTV-Flp transgene in the mammary gland and other organs, we obtained a *Rosa26*^*CAG-FSF-GFP*^ reporter strain that carries a targeted insertion of a CAG-FSF-GFP expression cassette in the endogenous *Rosa26* locus^29^. Since the reporter strain was originally maintained on a Swiss Webster × C57BL/6J mixed genetic background, we had to first transfer the *Rosa26*^*CAG-FSF-GFP*^ allele into the FVB genetic background by consecutively breeding mutant females with wildtype FVB/N males for five generations. In the absence of Flp recombinase, the *frt*-flanked transcriptional *STOP* sequence (FSF) located downstream of the CMV/chicken beta-actin promoter/enhancer (CAG) prevents the activation of the green fluorescent protein (GFP) (Fig. 2A). Following a temporally and spatially controlled activation of the recombinase, which excises the transcriptional *STOP* sequence, Flp-expressing cells and their descendants are genetically labeled with GFP. After crossing the reporter strain with the MMTV-Flp transgenic line (FVB/N), we examined the presence of GFP in selected organs of MMTV-Flp *Rosa26*^*CAG-FSF-GFP*^ double transgenic mice. Under a fluorescent stereoscope, the GFP expression from the *Rosa26*^*CAG-FSF-GFP*^ reporter in mammary gland tissues was low but detectable in most ducts (Fig. 2B). GFP was also present in selected areas of the salivary gland in both genders. Interestingly, the *Rosa26*^*CAG-FSF-GFP*^ reporter was not expressed in seminal vesicles, which is typically one of the main secretory organs in male mice that exhibit a strong activation of MMTV-LTR. Despite the detection of recombination events in the spleen and intestine by PCR (Fig. 1D), GFP-positive cells were not observed in any of these organs in MMTV-Flp *Rosa26*^*CAG-FSF-GFP*^ double transgenic mice, suggesting that the expression of the MMTV-Flp transgene is largely confined to the mammary gland and selected areas of the salivary gland.

The fluorescent co-staining of GFP along with cytokeratins 8 and 14 (CK8, CK14) revealed that the MMTV-Flp transgene is expressed in a subset of CK8-positive luminal epithelial cells within mammary ducts of nulliparous (virgin) mice and secretory alveoli of lactating females (Fig. 2C). We did not detect any regions in the mammary gland where the GFP reporter was active in CK14-expressing basal epithelial cells. In addition, GFP was not present in the mammary stroma, including fibroblasts, adipocytes, or lymphoid cells. Similar to the mammary gland, the MMTV-Flp is active in a subset of CK8-positive epithelial cells of the salivary gland and largely absent in CK5-expressing cells (Fig. 2D).

### MMTV-Flp-mediated activation of a ligand-inducible Cre recombinase in the normal mammary gland and during cancer development

**Figure 3:**
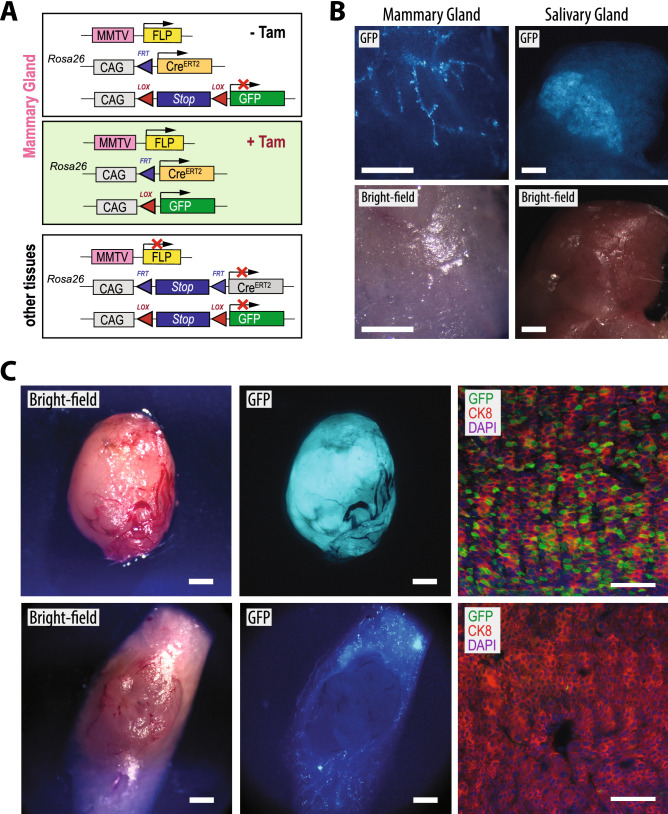
Ligand-inducible Cre recombinase action in an MMTV-Flp-dependent manner in normal and neoplastic mammary epithelial cells in vivo. (**A**) Schematic outline of the conditional expression of GFP from a Cre/*lox*P reporter transgene (CAG-LSL-GFP) following the tamoxifen (Tam)-inducible activation of the Cre^ERT2^ recombinase specifically in epithelial cells that express or that had expressed the MMTV-Flp transgene. (**B**) GFP and bight-field images of a mammary gland and salivary gland of an MMTV-Flp *Rosa26*^*CAG-FSF-CreERT2*^ CAG-LSL-GFP triple transgenic female; bars, 1 mm. (**C**) Matching bight-field and GFP fluorescent images (left and center) as well as immunofluorescent staining (right) of GFP-positive (upper) and GFP-negative (lower) mammary tumors from MMTV-neu MMTV-Flp *Rosa26*^*CAG-FSF-CreERT2*^ CAG-LSL-GFP quadruple transgenic females; bars, 1 mm (left, center) and 50 µm (right).

As highlighted in the “Introduction” section, a reason for developing the MMTV-Flp strain was to create experimental models that permit a ligand-controlled activation or deletion of genes at specific stages during normal development or cancer progression. For this purpose, we crossed the MMTV-Flp line with the *Rosa26*^*CAG-FSF-CreERT2*^ strain that expresses the tamoxifen (Tam)-inducible Cre recombinase (Cre^ERT2^) under the control of the CAG promoter following excision of the transcriptional *STOP* sequence (FSF) with Flp recombinase (Fig. 3A). To assess the activity of Cre^ERT2^ recombinase, we utilized a conventional CAG-LSL-GFP transgene that, as we have demonstrated previously, exhibits a very high expression of GFP in the normal mammary gland and mammary cancers of MMTV-Cre and WAP-Cre transgenics^25,28,30,31^. Nulliparous and nonpregnant parous MMTV-Flp *Rosa26*^*CAG-FSF-CreERT2*^ CAG-LSL-GFP triple transgenic females were treated with tamoxifen (Tam) as described in the “Methods” section, and tissues were collected 2 to 4 weeks later. Although GFP-positive ducts could be detected in unfixed specimens of the mammary gland and parts of the salivary gland (Fig. 3B), the overall recombination efficiency throughout the ductal tree was inferior compared to the activation of GFP that we normally see in MMTV-Cre CAG-LSL-GFP females, possibly due to the mosaic expression of the MMTV-Flp^25,28^. These findings were likely not a consequence of the experimental procedures associated with the administration of Tam, the examination of the tissues, or the genetic strain background. Pdx-Flp *Rosa26*^*CAG-FSF-CreERT2*^ CAG-LSL-GFP mice that are being maintained in our colony show a uniform activation of GFP in response to the same tamoxifen treatment regimen as reported earlier^26^. GFP-positive mammary glands were never observed in control mice that had not received Tam or animals that did not carry the MMTV-Flp transgene (not shown).

To determine whether it was technically feasible to activate or to delete genes in a tamoxifen-inducible and Cre-mediated manner in mammary cancers, we generated parous female mice that carried the MMTV-neu oncogene in addition to the MMTV-Flp *Rosa26*^*CAG-FSF-CreERT2*^ CAG-LSL-GFP transgenes. Quadruple transgenic females were repeatedly bred to accelerate the formation of MMTV-neu-induced tumors. Nonpregnant parous mice were treated with Tam prior to or immediately after tumors became palpable. Tissues were collected before the tumors had reached a size of 1 cm in diameter. A bright fluorescent signal throughout an entire mammary tumor was indicative that the MMTV-Flp transgene was active and that the recombined *Rosa26*^*CAG-FSF-CreERT2*^ was functional in ERBB2/neu-expressing mammary tumor cells in vivo (Fig. 3C, upper panels). In these tumors, the GFP signal was detected in CK8-positive cancer cells that represent most tumor cells in this luminal-type breast cancer model. We also observed mammary tumors that exhibited a complete absence of GFP despite a widespread activation of the reporter in normal or preneoplastic mammary ducts adjacent to the large tumor (Fig. 3C, lower panels). This phenomenon was also seen in Tam-treated animals with multiple tumors that were GFP-positive or GFP-negative, which excludes the possibility of experimental variations in Tam administration. This finding suggested that both MMTV-driven transgenes exhibited a mosaic expression pattern and that the MMTV-Flp was not always active in the same cell-of-origin that gave rise to an ERBB2-induced tumor. Therefore, we highly recommend using a Cre/lox reporter transgene as part of the experimental model to monitor the synergistic functionality of both recombinases within individual tumors.

### The MMTV-Flp-mediated expression of mutant KRAS from its endogenous gene results in metastatic mammary cancer

**Figure 4:**
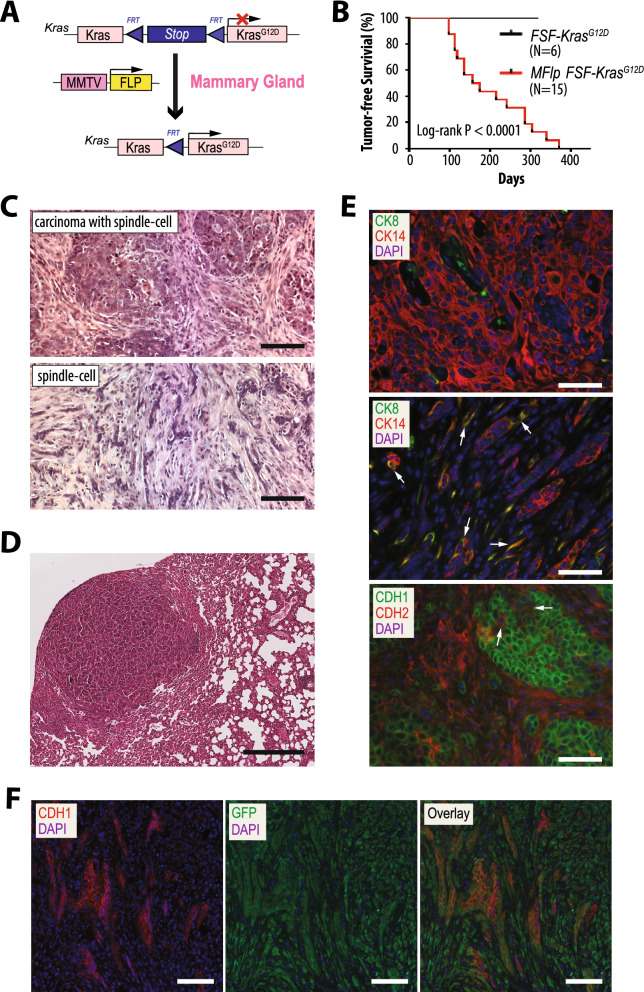
Initiation of metastatic mammary cancer in response to the MMTV-Flp-induced expression of mutant KRAS from its endogenous locus. (**A**) Graphic illustration of the conditional activation of an oncogenic *Kras*^*G12D*^ allele in response to the expression of the MMTV-Flp transgene in the mammary epithelium. (**B**) Kaplan–Meier survival plot of double transgenic MMTV-Flp *FSF-Kras*^*G12D*^ female mice in comparison to littermate *FSF-Kras*^*G12D*^ single mutant controls. Please note that in the absence of the Flp recombinase, the *FSF-Kras*^*G12D*^ allele is a functional knockout of KRAS. The statistical significance in tumor-free survival between the controls and experimental animals was calculated with the log-rank (Mantel-Cox) test. (**C,D**) Hematoxylin and eosin-stained histological sections of mammary cancers (**C**) and pulmonary metastasis (**D**) in mice expressing oncogenic KRAS; bars, 100 µm (**C**) and 400 µm (**D**). (**E**) Immunofluorescent (IF) staining of cytokeratins (CK) 8 and 14 in mammary tumors with epithelial (upper) and mesenchymal-like (middle) characteristics as well as E- and N-cadherin (CDH1, CDH2) in a poorly differentiated mammary tumor with mixed cellular features (lower); bars, 50 µm. Arrows point to cancer cells that have retained CK8/14 co-expression (middle) or those that exhibit epithelial morphology but express N-cadherin (lower). (**F**) IF staining of E-cadherin (CDH1) and GFP in a mammary tumor that originated in a MMTV-Flp *FSF-Kras*^*G12D*^* Rosa26*^*CAG-FSF-GFP*^ triple transgenic female; bars, 50 µm. The *Rosa26*^*CAG-FSF-GFP*^ was used in this model to genetically label the fate of MMTV-Flp-expressing cells that gave rise to tumors in response to the Flp-induced activation of oncogenic KRAS. Please note that KRAS^G12D^ and GFP are constitutively expressed in a differentiation-independent manner regardless of whether transdifferentiating cancer cells retain or lose the expression of the MMTV-Flp transgene or epithelial characteristics.

To determine whether the MMTV-Flp transgenic strain could serve a dual purpose for the initiation of mammary tumors and the subsequent deletion or activation of genes in a ligand-controlled manner, we developed a mammary cancer model that expresses oncogenic KRAS in response to the MMTV-driven expression of Flp in the mammary gland. First, we backcrossed the *Kras*^*G12D*^ knockin (*FSF-Kras*^*G12D*^) allele into the FVB/N background for seven generations and mated the strain to the MMTV-Flp transgenic line to generate double transgenic females (Fig. 4A). Palpable mammary tumors were detected in MMTV-Flp *FSF-Kras*^*G12D*^ double transgenic females at a median latency of 159 days (Fig. 4B). In contrast to other models where pregnancy greatly accelerates the development of mammary cancer, the latency of mammary tumor formation in the mutant KRAS-expressing females was not significantly affected by parity (165 days, N = 10, in virgin versus 136 days, N = 9, in parous mice; *P* > 0.2). It is interesting to note that despite the expression of the MMTV-Flp in the salivary gland of both genders, we did not detect any palpable tumors in this tissue, particularly in male breeders that were kept for up to one year. Without the Flp recombinase, the *FSF-Kras*^*G12D*^ allele is a functional knockout of KRAS, and consequently, mammary tumors and other malignancies were not observed in any of the single-transgenic littermates.

On the histological level, mammary tumors from MMTV-Flp *FSF-Kras*^*G12D*^ females were poorly differentiated adenocarcinomas with extensive areas of spindle cells (Fig. 4C), and larger tumors were mostly comprised of spindle-type cancer cells. At the time of necropsy, when tumors had reached approximately 1.5 cm in diameter, metastatic lesions were detected in the lungs (Fig. 4D). Given that the expression of the MMTV-Flp transgene was restricted to CK8-positive luminal epithelial cells of the normal mammary gland (Fig. 2C), it was surprising that neoplastic cells in poorly differentiated adenocarcinomas expressed the basal epithelial cell marker CK14 (Fig. 4E, upper). Spindle-cell carcinomas consisted of mesenchymal-like cells with significantly reduced levels of cytokeratins (Fig. 4E, middle). The dual staining of poorly differentiated adenocarcinomas for E- and N-cadherin revealed that similar to the spindle-shaped cells, a subset of carcinoma cells with typical epithelial morphology expressed N-cadherin (Fig. 4E, lower, arrows). These observations suggest that, in addition to promoting the occurrence of basal-like mammary cancers, the expression of oncogenic KRAS facilitates cellular plasticity towards a mesenchymal phenotype. This notion is supported by evidence from the genetic labeling of KRAS^G12D^-expressing cancer cells with the Flp-inducible *Rosa26*^*CAG-FSF-GFP*^ reporter (Fig. 4F). Poorly differentiated adenocarcinoma cells that retained expression of E-cadherin (CDH1), as well as CDH1-negative spindle-like cells within mammary tumors of MMTV-Flp *FSF-Kras*^*G12D*^* Rosa26*^*CAG-FSF-GFP*^ triple transgenic females, were GFP-positive. Considering that the MMTV-Flp is not expressed in stromal cells of the mammary gland (Fig. 2C), it is evident that the GFP-positive mesenchymal-like cells are descendants of transformed luminal epithelial cells and not cancer-associated fibroblasts.

### Mutant p53 accelerates the onset and malignant progression of KRAS-induced mammary cancer

**Figure 5:**
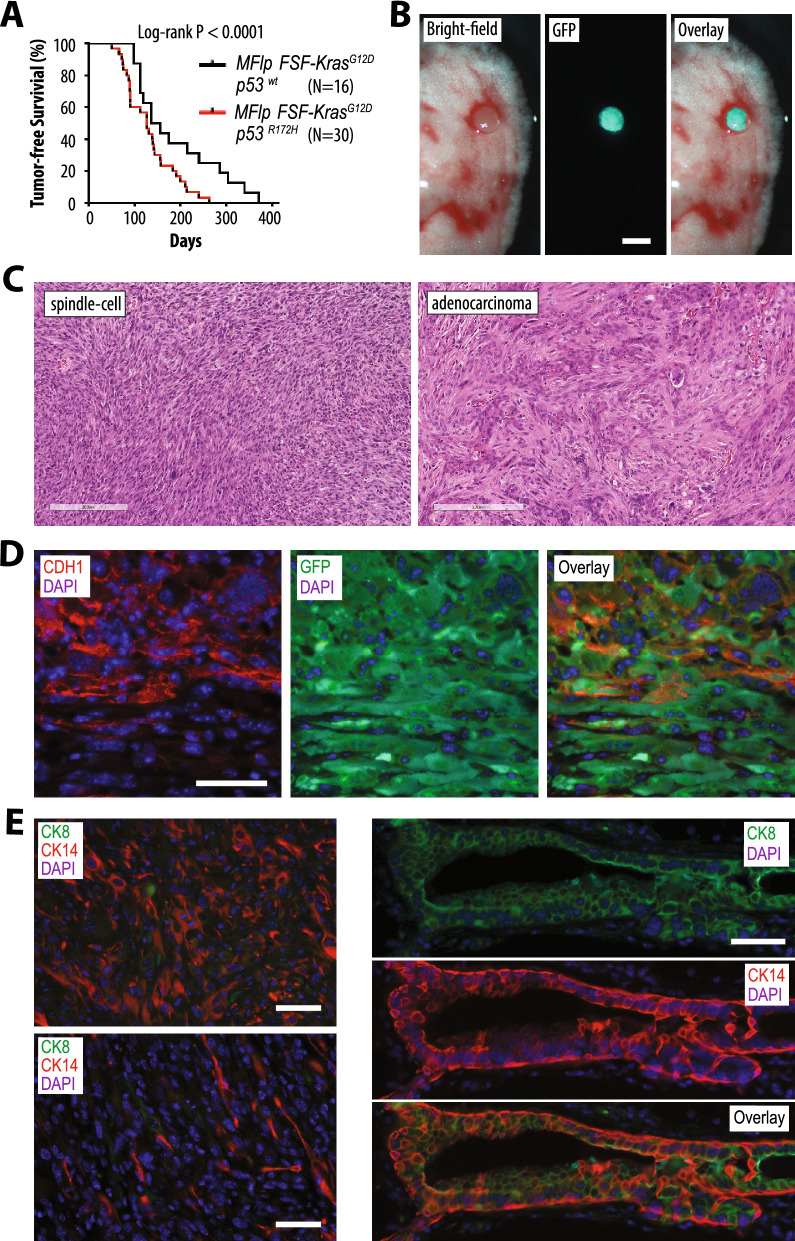
A single mutant *p53*^*R172H*^ allele leads to an earlier onset and faster malignant progression of KRAS-induced mammary cancer. (**A**) Kaplan–Meier plot comparing the tumor-free survival of females that expresses KRAS^G12D^ in the mammary gland (MMTV-Flp FSF-*Kras*^*G12D*^) with or without one mutant *p53*^*R172H*^ allele. Statistical significance in tumor-free survival between these experimental groups was calculated with the log-rank (Mantel-Cox) test. (**B**) Bight-field and GFP fluorescent images of the lung tissue with a GFP-positive metastatic lesion from a mammary tumor-bearing MMTV-Flp *FSF-Kras*^*G12D*^* Rosa26*^*CAG-FSF-GFP*^* p53*^*R172H*^ quadruple transgenic female; bars, 1 mm. (**C**) Hematoxylin and eosin-stained histological sections of mammary cancers from MTV-Flp *FSF-Kras*^*G12D*^* p53*^*R172H*^ mice; bars, 200 µm. (**D**) Immunofluorescent (IF) staining of E-cadherin (CDH1) and GFP in a mutant *p53*^*R172H*^ and KRAS^G12D^-induced mammary tumor; bar, 50 µm. (**E**) IF staining of cytokeratins (CK) 8 (green) and 14 (red) in mammary tumors with predominantly epithelial or mesenchymal appearance (left) and a premalignant mammary duct (right); bars, 50 µm.

The majority of basal-like and claudin-low (i.e., mesenchymal-like) breast cancers in humans carry mutations in *Trp53*. The inclusion of the mutant *p53*^*R172H*^ allele significantly shortened the median survival of MMTV-Flp *FSF-Kras*^*G12D*^ females by approximately 1 month (Fig. 5A; 127 days, N = 30, in *p53*^*R172H*^ mice versus 159 days, N = 19, in *p53*^*wt*^ females; *P* < 0.0001). Palpable mammary tumors were detected as early as 51 days of age in females that had not been pregnant. To monitor the occurrence of lung metastases in this breast cancer model, we generated eight MMTV-Flp *FSF-Kras*^*G12D*^* p53*^*R172H*^ female mice that also carried the *Rosa26*^*CAG-FSF-GFP*^ reporter. All mammary tumor-bearing MMTV-Flp *FSF-Kras*^*G12D*^* p53*^*R172H*^* Rosa26*^*CAG-FSF-GFP*^ quadruple transgenics had metastatic lesions in their lungs at the time of necropsy (Fig. 5B).

In comparison to the mammary tumors in the wildtype p53 background, *FSF-Kras*^*G12D*^-induced cancers with the *p53*^*R172H*^ mutant allele were mostly comprised of mesenchymal-like spindle cells, and fewer tumors were classified as poorly differentiated adenocarcinomas (Fig. 5C). This suggested that mutant p53 not only accelerated the onset of mammary cancer but also promoted a swift progression for tumors to acquire mesenchymal-like characteristics. The activity of the GFP reporter in tumor cells irrespective of E-cadherin expression status was indicative that all cancer cells, including those with mesenchymal features, had originated from MMTV-Flp-expressing luminal epithelial cells (Fig. 5D). This notion was supported by the observation that CK8-positive luminal epithelial cells within preneoplastic mammary ducts co-expressed CK14, which is normally only present in basal epithelial cells (Fig. 5E, left). The resulting poorly differentiated mammary cancers contained mostly CK14-positive tumor cells, and the cytokeratin expression was progressively lost in mesenchymal-like tumors (Fig. 5E, right).

### Combinatorial action of the Flp and Cre recombinases in KRAS-driven mammary cancers

**Figure 6:**
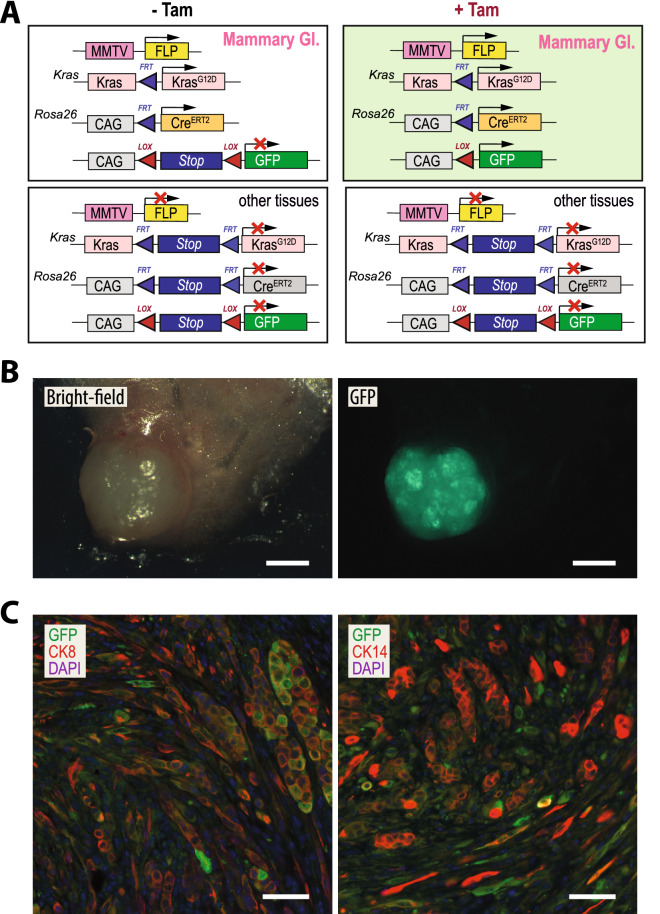
Testing the dual functionality of the Flp and Cre recombinases in KRAS-driven mammary cancers. (**A**) Diagram of the MMTV-Flp/Cre^ERT2^-codependent expression of GFP from a Cre/loxP reporter transgene (CAG-LSL-GFP) in KRAS^G12D^-induced mammary tumors in response to the treatment with tamoxifen (Tam). (**B**) Bight-field and GFP fluorescent images of a mammary tumor from an MMTV-Flp *FSF-Kras*^*G12D*^* Rosa26*^*CAG-FSF-CreERT2*^ CAG-LSL-GFP quadruple transgenic female that was treated with Tam; bar, 2 mm. (**C**) Immunofluorescent staining of GFP (green) along with cytokeratins (CK) 8 or 14 (both red) in mammary tumor sections of a Tam-treated quadruple transgenic female; bars, 50 µm.

After establishing the Flp recombinase-based mammary cancer model expressing mutant KRAS, we embarked on a study to determine whether it was technically possible that Flp can serve a dual purpose for the subsequent activation or deletion of genes in established mammary tumors. We generated female mice that carried the MMTV-Flp and *FSF-Kras*^*G12D*^ in combination with the Flp-inducible *Rosa26*^*CAG-FSF-CreERT2*^ allele and the CAG-LSL-GFP reporter transgene (Fig. 6A). In contrast to the combination of the MMTV-Flp with the MMTV-neu oncogene that are not always co-expressed in the same cancer cell-of-origin (Fig. 3), we assumed that MMTV-Flp-induced mammary tumors expressing oncogenic KRAS should also carry a Flp-activated *Rosa26*^*CAG-FSF-CreERT2*^ allele that would subsequently facilitate the tamoxifen (Tam)-controlled activation of Cre recombinase and expression of GFP from the Cre/lox reporter transgene. Since oncogenic KRAS causes triple-negative mammary tumors, a short administration of Tam is not expected to have any biological effect on the growth and survival of mammary cancer cells. Following the detection of mammary tumors, MMTV-Flp *FSF-Kras*^*G12D*^* Rosa26*^*CAG-FSF-CreERT2*^ CAG-LSL-GFP quadruple transgenic females were treated with Tam for five consecutive days. Tumors were collected 2 weeks later and examined with a fluorescent stereoscope (Fig. 6B). A bright fluorescent signal in the tumors was evidence that Flp recombinase was able to coactivate the cancer-inducing *FSF-Kras*^*G12D*^ allele as well as the *Rosa26*^*CAG-FSF-CreERT2*^ that targeted the expression of a functional Cre^ERT2^ fusion protein to the cancer cells. The histological examination of GFP expression in cancer cells showed that the Cre^ERT2^ fusion protein was active in a subset of cells within tumors that had retained an expression of CK8 (Fig. 6C, left) as well as the majority of cancer cells that acquired basal and mesenchymal-like characteristics, i.e., those that were CK14-positive or lacked cytokeratin expression (Fig. 6C, right). Since the MMTV-Flp was not expressed in the mammary stroma, the presence of GFP is the result of the combinatorial activities of the Flp and Cre recombinases in the cancer cell-of-origin and their descendants even if they lose the expression of the MMTV-Flp during metaplastic progression. In principle, this experimental approach can be viewed as an additional cell lineage-tracing experiment that provides supporting evidence that the mesenchymal-like cells are metaplastic tumor cells that originated from Flp-expressing luminal epithelial cells and are not cancer-associated fibroblasts.

### A ligand-controlled deletion of JAK1 in KRAS-induced mammary cancer cells blocks the oncogenic activation of STAT3

**Figure 7:**
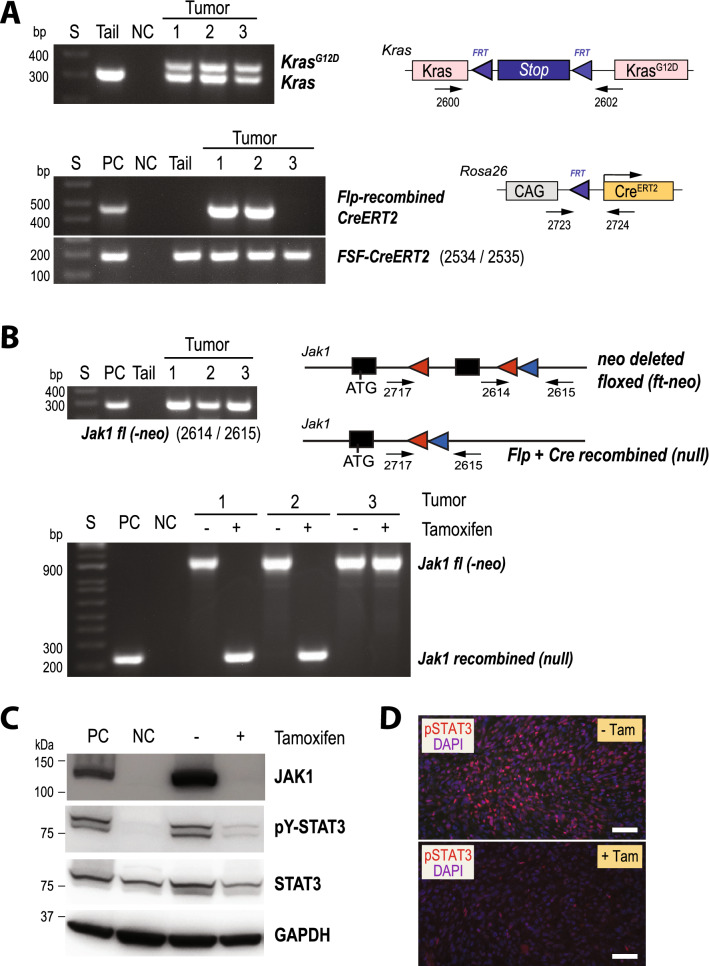
The JAK1 tyrosine kinase controls the oncogenic activation of STAT3 in KRAS-induced mammary cancer cells. (**A**) PCR assays to validate the MMTV-Flp-mediated activation of the *FSF-Kras*^*G12D*^ allele (upper panel) and the *Rosa26*^*CAG-FSF-CreERT2*^ allele (lower panel) in mammary tumor cells from MMTV-Flp *FSF-Kras*^*G12D*^* Rosa26*^*CAG-FSF-CreERT2*^* Jak1*^*fl/fl*^ females. DNA from the tail of a mammary tumor-bearing animal was used as a control (upper panel). DNA from cells of a Pdx-Flp-induced pancreatic cancer that carried the *Rosa26*^*CAG-FSF-CreERT2*^ allele was used as a positive control (PC) for the *FSF* recombination in the *Cre*^*ERT2*^ allele; *NC* no DNA, *S* DNA size markers. (**B**) Upper panel: PCR to determine the Flp-mediated deletion of the *frt*-flanked neomycin selection cassette in the *Jak1* conditional knockout alleles of the tumor cells shown in (**A**). Lower panel: PCR assay to assess the Cre^ERT2^-mediated deletion of the second coding exon of *Jak1* before and after treatment with tamoxifen (Tam). DNA from MEFs with defined Flp and Cre-mediated deletions were used as positive controls (PC), and tail DNA from panel A served as negative control (NC). (**C**) Western blot analysis of mammary tumor cells before and after treatment with Tam to assess the effects of a Cre^ERT2^-mediated deletion of JAK1 on STAT3 activation. GAPDH served as a loading control. A pair of isogenic cells from ERBB2-induced mammary tumors with JAK1 (PC) and without JAK1 (NC) was used as controls. (**D**) Immunofluorescent staining of pSTAT3 in histological sections of secondary mammary tumors that resulted from the transplantation MMTV-Flp *FSF-Kras*^*G12D*^* Rosa26*^*CAG-FSF-CreERT2*^* Jak1*^*fl/fl*^ mammary tumor cells and treatment with or without Tam.

Finally, we performed a proof-of-principle experiment to assess whether it is possible to effectively delete two copies of an endogenous gene in mammary cancer cells in a ligand-controlled manner as a result of the combined action of the two recombinases. For this purpose, we used the conditional *Jak1* knockout that was employed earlier to monitor the activation of Flp (Fig. 1) to create a knockout (null) allele of the Janus kinase 1 with Cre recombinase in a Tam-dependent manner (Fig. 7). MMTV-Flp *FSF-Kras*^*G12D*^* Rosa26*^*CAG-FSF-CreERT2*^* Jak1*^*fl/fl*^ females were bred and monitored weekly for tumor onset. To systematically assess every step of the dual recombinase action, we isolated mammary cancer cells from three tumors that were approximately 1 cm in size. The expected Flp-mediated activation of the cancer-inducing *FSF-Kras*^*G12D*^ allele was validated by PCR (Fig. 7A, upper). The equal ratio of the amplification products between the activated mutant and wildtype *Kras* alleles is indicative of the purity of the derived cancer cells. It should be noted that all tumor-bearing animals were heterozygous for the *FSF-Kras*^*G12D*^ since this targeted insertion is a functional null allele of KRAS that causes embryonic lethality in a homozygous configuration. The analysis of the Flp-mediated recombination event in the *Rosa26*^*CAG-FSF-CreERT2*^ knockin revealed that the Flp recombinase accurately deleted the *FSF* cassette in two of the three tumors (Fig. 7A, lower). This was an unexpected finding since the Flp recombinase was able to delete the *frt*-flanked neomycin cassette in the conditional *Jak1* knockout alleles in addition to activating the *FSF-Kras*^*G12D*^ that led to tumor formation (Fig. 7B, upper). Upon treatment with Tam, the ligand-inducible Cre^ERT2^ recombinase facilitated the conversion of the floxed *Jak1* into a knockout allele in the two cancer cell lines where Flp had correctly recombined the *Rosa26*^*CAG-FSF-CreERT2*^ locus (Fig. 7B, lower). A preliminary analysis showed that one of the cancer cell lines with all correctly targeted alleles had a high expression of active (i.e., tyrosine phosphorylated) STAT3. The immunoblot analysis of the isogenic cell line pair with and without Tam revealed that the ligand-inducible knockout of JAK1 was very efficient and resulted in the deactivation of its downstream target STAT3 (Fig. 7C). Tyrosine phosphorylated STAT3 was also significantly reduced in secondary tumors in vivo that originated in wildtype recipient females from the transplantation of MMTV-Flp *FSF-Kras*^*G12D*^* Rosa26*^*CAG-FSF-CreERT2*^* Jak1*^*fl/fl*^ mammary tumor cells and treatment with Tam (Fig. 7D). In summary, this line of investigation demonstrated that a ligand-controlled deletion of target genes can be accomplished in mammary cancer cells using an experimental approach with two recombinases. However, a systematic analysis of each step in this process needs to be monitored to assess the precision of the actions of both recombinases on their respective target genes. Finally, this proof-of-principle study provides evidence that the JAK1 tyrosine kinase plays a central role in the constitutive activation of STAT3 in KRAS-transformed mammary cancer cells.

## Discussion

Over the past three decades, various MMTV-LTRs that differ in length and origin were used as promoters to target the expression of genes to the mammary gland of transgenic mice^2^. The activation of a shorter MMTV-LTR variant in the mammary gland seems to be less dependent on the genetic strain background, but this promoter is expressed in many other tissues^32,33^. In contrast, a longer MMTV-LTR has been shown to contain regulatory elements that permit a more stringent expression of exogenous proteins in the mammary gland^34–36^. Because of its superior tissue specificity, we had previously used the extended MMTV-LTR to target the tetracycline-controlled transactivator (tTA) to the mammary epithelium^27^. We observed that the MMTV-tTA transgene was expressed in the perinatal mammary gland and could be used for gender identification of newborn pups when they carried the transgene along with a TetO-H2B-GFP reporter. In a recent study, we validated that the tTA under the control of the extended MMTV-LTR is mainly expressed in luminal epithelial cells of the mammary gland in postnatal and adult females^37^. The identical promoter and intronic/polyA sequences of the MMTV-tTA were used to construct the MMTV-Flp transgene. Using the *Rosa26*^*CAG-FSF-GFP*^ reporter strain, we determined that the codon-optimized Flp recombinase under the control of the extended MMTV-LTR was stringently expressed, albeit in a mosaic fashion, in luminal epithelial cells of adult females. Since the Flp-mediated recombination leads to an irreversible activation of the GFP reporter in a cell and all of its descendants, our observation suggests that the MMTV-Flp is silent in basal epithelial cells or bi-potent progenitors in the embryonic or postnatal mammary gland. Overall, the expression profile of our new transgenic strain is similar to the MMTV-Flpo line 9 generated recently by Luond et al.^38^. Expression of Flp in mesenchymal cells of the mammary stroma was never observed. Since Flp was functional in luminal-type mammary tumors of MMTV-PyMT^38^ and MMTV-neu (Fig. 3) transgenic females, the MMTV-Flp transgenic lines might be suitable genetic tools for cell lineage tracing and subsequent manipulation of target genes in neoplastic cells.

To demonstrate the applicability of the new MMTV-Flp strain to model breast cancer, we used this transgene for the recombinase-induced activation of an oncogenic mutant of KRAS that is expressed from its endogenous locus. We show here that the expression of mutant KRAS in a p53 wildtype background was sufficient to initiate the development of poorly differentiated mammary cancers that possess the propensity to metastasize. The inclusion of a mutant *p53*^*R172H*^ allele led to an accelerated onset of oncogenic KRAS-induced tumor formation and a more frequent occurrence of tumors with predominantly mesenchymal-like features. As part of a comprehensive project on the roles of oncogenic RAS signaling in cellular plasticity, we conducted a thorough molecular and cellular examination of the poorly differentiated and mesenchymal-like mammary cancers that originated in MMTV-Flp *FSF-Kras*^*G12D*^* p53*^*R172H*^ females^37^. According to their genome-wide gene expression profiles and validated protein markers, the tumor with the two distinctly different histopathological features belong to the basal-like and claudin-low molecular subtypes of triple-negative mammary cancer. In this new study, we used a reporter allele for the Flp recombinase as a cell lineage-tracing tool to demonstrate that luminal epithelial cells in MMTV-Flp *FSF-Kras*^*G12D*^ females acquire characteristics of basal epithelial cells and gave rise to basal-like mammary tumors that progressively gain mesenchymal features. We showed that the MMTV-LTR is not expressed in the stroma of the mammary gland and, therefore, the spindle-shaped cells with mesenchymal features are cancer cells and not tumor-associated fibroblasts. This is the same developmental trajectory of mammary cancers that we observed in WAP-Cre EF1-LSL-tTA TetO-Kras^G12D^ transgenic females expressing exogenous KRAS^G12D^ in luminal epithelial cells^37^. Once activated in the luminal epithelium, the expression of exogenous or endogenous mutant KRAS occurs in a differentiation-independent manner in both mammary cancer models, which is a prerequisite to uncover the full extent of cellular plasticity that is mediated by the oncogenic driver. The collective findings from our studies support the notion from recent bioinformatics analyses that hyperactive RAS signaling is a recurrent feature across all claudin-low breast cancers in humans^39,40^. We also recently demonstrated that the constitutive expression of oncogenic KRAS persistently upholds the mesenchymal characteristics of claudin-low tumor cells. Following the downregulation of the oncogenic driver, claudin-low mammary tumor cells regain the expression of luminal epithelial cell lineage markers^37^. It will be interesting to see whether the deletion of the Flp-induced endogenous mutant *Kras* allele will also result in the reversal of the developmental trajectory of claudin-low cancer cells.

Besides using the MMTV-Flp transgene to activate the expression of a mammary cancer-inducing oncogene and to trace basal and mesenchymal-like tumors back to their luminal epithelial cell origin, we tested the applicability of the transgenic line to activate or delete genes in a ligand-controlled manner based on the combinatorial action of two recombinases. For this purpose, we employed the targeted *Rosa26*^*CAG-FSF-CreERT2*^ allele that expresses the tamoxifen-inducible Cre^ERT2^ fusion protein in a Flp recombinase-dependent manner^26^. The ability of the MMTV-Flp to activate the *Rosa26*^*CAG-FSF-CreERT2*^ allele and the downstream expression of a GFP-based Cre/lox reporter transgene in a ligand-controlled manner was tested in three experimental models: the normal mammary gland, luminal-type mammary tumors that overexpress ERBB2, and the new KRAS^G12D^-induced mammary cancer model in which tumor cells acquire basal-like and mesenchymal characteristics. The collective results from these various lines of investigation showed that it is, in principle, feasible to manipulate genes in a tamoxifen-inducible manner in normal and neoplastic mammary epithelial cells, even after tumor cells have become basal or mesenchymal-like. Due to the mosaic expression pattern of the MMTV-Flp, the dual recombinase approach might be less suitable for a complete deletion of genes in most luminal cells of the normal mammary gland ductal tree. To excise genes from progressing mammary cancers, it is important to confirm that the MMTV-Flp was active in the cell(s)-of-origin that gave rise to these tumors. While this is an obvious requirement for cancers that are being caused by other MMTV promoter-driven transgenes, it is also recommended to monitor the functionality of the Flp recombinase on all essential target alleles that contain *frt* sites in cancer cells that undergo neoplastic transformation and a mesenchymal transition soon after the MMTV-Flp is expressed. This might be particularly relevant when these cellular processes are being accelerated in the backdrop of mutant p53.

The relatively speedy development of basal and claudin-low primary mammary cancers and the high propensity of these tumors to disseminate to distant sites make the MMTV-Flp *FSF-Kras*^*G12D*^ transgenic mice a particularly useful model to study the molecular mechanisms of tumor initiation, metastatic progression, and cellular plasticity. The persistent activation of STAT3 in response to Il-6 class inflammatory cytokines is a hallmark of many early-stage and advanced human malignancies. Recent work from our team has provided evidence that the Janus kinase 1 (JAK1), and not JAK2 as generally believed, is the tyrosine kinase that plays a pivotal role in activating STAT3 in normal and neoplastic mammary epithelial cells^25,28^. In a proof-of-principle experiment, we used the dual recombinase approach in this study to conditionally delete JAK1 in KRAS-transformed mammary cancer cells. The results of this feasibility study show that it is possible to delete both alleles of *Jak1* in cancer cells with high efficiency in a tamoxifen-inducible manner. The absence of JAK1 in this triple-negative mammary cancer model was sufficient to block the tyrosine phosphorylation of STAT3, suggesting JAK1 is a suitable therapeutic target to uncouple the oncogenic activation of STAT3 from its upstream inflammatory cytokines.

## Methods

### Cloning of the MMTV-Flp transgene

The coding sequence of the codon-optimized Flp recombinase (FLPo) that contains a nuclear localization signal (NLS) was synthesized by IDT, Inc. based on sequence information from the pQUAST-FLPo construct (Addgene # 24357). The inclusion of *Hin*dIII and *Eco*RI restriction sites surrounding the NLS-FLP with a preceding Kozak consensus sequence permitted a directional subcloning of the recombinase fragment into the corresponding *Hin*dIII and *Eco*RI sites located between the MMTV-LTR and the SV40 small t intron and polyA signal of a modified MMTV-SV40-BSSK vector^27^. The original MMTV-SV40-BSSK vector was a kind gift from Dr. Muller at McGill University^41^. The complete transgene was sequenced.

### Generation of MMTV-Flp transgenic mice and genotyping protocol

The MMTV-Flp transgene was released from the vector as a *Spe*I fragment of 5.27 kb in size and injected into FVB zygotes. The pronuclear injection and generation of founder mice were performed at Cyagen US, Inc. The integration of the transgene into the genome was determined by PCR using primers that amplify a 344 bp fragment at the Flp/SV40 intron small t intron junction (5′-ATC GAG GAG TGG CAG CAC ATC G-3′ and 5′-CAC TGC TCC CAT TCA TCA GTT CCA-3′). Using this method, four transgenic founder animals (two males and two females) were identified. The four founders (29539, 29540, 29541, and 29542) were mated with FVB/N wildtype mice to determine the germline transmission of the transgene and to establish individual transgenic lines. All mice were fertile and produced multiple litters, but female founder 29541 did not transmit the transgene to her offspring. Routine genotyping of MMTV-Flp transgenic mice was carried out by PCR using primers corresponding to the MMTV-LTR and the *Flp* coding sequence (2127; 5′-AGT GAT AGA GCT CTT GCC TAG C-3′ and 2525; 5′-CGT TGT AAG GGA TGA TGG TGA ACT-3′). Alternatively, we used a primer set that detects the 3′ end of *Flp* and the SV40 intron/polyA sequence (2546; 5′-CCT GGA ACG GCA TCA TCA GC-3′ and 2128; 5′-CTC CCA TTC ATC AGT TCC ATA GG-3′). The resulting PCR fragments were 570 bp and 275 bp in size, respectively. After assessing the transgene expression patterns of the three lines that had transmitted the MMTV-Flp construct, the founder line 29542 was used in subsequent functional studies and was registered at the Mouse Genome Informatics (MGI) Database as Tg(MMTV-Flp)29542Kuw (MGI:6392338). Further information and requests for MMTV-Flp transgenic mice should be directed to the Lead Contact, Kay-Uwe Wagner (kuwagner@wayne.edu).

### Additional genetically modified mouse strains and procedures

The generation of JAK1 conditional knockout mice with *loxP* sequences flanking the second coding exon was reported earlier^25,42^. The *Jak1* conditional knockout allele (*Jak1*^*fl*^) in these mice contained the PGK-neomycin selectable marker in intron 3 that is surrounded by *frt* sequences. A detailed description about the *Rosa26*^*CAG-FSF-CreERT2*^ knockin strain expressing a tamoxifen-inducible Cre recombinase in a Flp-dependent manner [MGI:5616874; *Gt(ROSA)26Sor*^*tm3(CAG-Cre/ERT2)Dsa*^] was published by Schönhuber et al.^26^. The CAG-LSL-GFP transgenic reporter strain that expresses GFP in a Cre-dependent manner was kindly provided by Dr. Miyazaki (Osaka University)^30^. Mice expressing the wildtype ERBB2 (Her2/neu) receptor under the MMTV-LTR (MMTV-neu) were generated by Guy et al.^34^. The mouse strains that carry the targeted alleles of the *FSF-Kras*^*G12D*^ [B6(Cg)-Kras^tm5Tyj^/J]^43^ and the *Rosa26*^*CAG-FSF-GFP*^ [Gt(ROSA)26Sor^tm1.2(CAG-EGFP)Fsh^]^29^ were kind donations from the laboratories of Drs. Jacks and Fishell to The Jackson Laboratory (Stock No: 023590 and 32038-JAX, respectively). The *Trp53*^*R172H*^ allele was created by crossing mice with the *p53*^*LSL-R172H*^ targeted locus [B6.129S4-Trp53^tm2Tyj^/Nci; Strain No: 01XAF at the NCI Mouse Repository]^44^ with MMTV-Cre line A (FVB/N) transgenics^6^, and by subsequently mating double mutant females with wildtype FVB/N males to segregate out the MMTV-Cre and to transmit the germline recombined *Trp53*^*R172H*^ allele to the offspring. MMTV-neu, MMTV-Cre, and MMTV-Flp mice were originally generated in the FVB strain, and CAG-LSL-GFP transgenics and the JAK1 conditional knockout allele were maintained on this background for more than 20 generations^28^. For this project, the following targeted alleles were backcrossed for the indicated number of generations (N) into the FVB/N genetic background: *Trp53*^*R172H*^ (N = 7), *FSF-Kras*^*G12D*^ (N = 7), *Rosa26*^*CAG-FSF-CreERT2*^ (N = 5), and the *Rosa26*^*CAG-FSF-GFP*^ (N = 5). Supplementary Table 1 lists all PCR primers that were used for genotyping and for assessing the Flp and Cre-mediated recombination events. We found that a combination of primers 2729, 2730, and 2731 in a PCR reaction can be used to discriminate heterozygous or homozygous configurations or specific combinations of the *Rosa26*^*CAG-FSF-CreERT2*^, *Rosa26*^*CAG-FSF-GFP*^, and the wildtype *Rosa26* alleles. Mammary tumor cells were injected orthotopically into the #4 inguinal mammary glands of 12-week-old female wildtype NCr^nu/nu^ recipients as described^23^. To induce a Cre^ERT2^-mediated deletion of the JAK1 conditional knockout alleles or to activate the CAG-LSL-GFP reporter, mice were treated with tamoxifen (Tam) once daily for 5 consecutive days^26^. All mice were housed on a 12/12-h light/dark cycle in pathogen-free micro-isolator cages. The care and use of animals followed the ARRIVE guidelines, and this work was conducted in accordance with the recommendations in the Guide for the Care and Use of Laboratory Animals of the National Institutes of Health. The animal study protocols were approved by the Institutional Animal Care and Use Committee of the University of Nebraska Medical Center and Wayne State University (protocols 03-104-01-FC, 09-104-01-FC, 12-008-03-FC, 19-07-1190, and 19-07-1196).

### Histological analysis and immunofluorescent staining

Unfixed specimens of the thoracic and inguinal mammary glands and other organs were mounted on glass slides and examined with a Discovery.V8 fluorescence stereoscope (Carl Zeiss, Inc.) for GFP expression. For subsequent histological examination and immunostaining procedures, tissues were fixed overnight in 10% buffered formalin (Fisher Scientific Company) and stored in 70% ethanol prior to paraffin embedding. Histologic sections of 5 µm were deparaffinized three times in 100% Histo-Clear, rehydrated in decreasing concentrations of ethanol (100%, 95%, 90%, 70%, 50%, and 30% for 3 min each), and washed for 5 min in 1× PBS. Slides were stained with Hematoxylin and Eosin (H&E) for routine histology. For immunofluorescent staining, tissue sections were pressure cooked in ImmunoRetriever with Citrate (Bio SB) using a Bio SB TintoRetriever pressure cooker (116–121 °C for 4 min). The slides were cooled to room temperature, rinsed for 5 min in 1× PBS, and blocked with 3% BSA for 1 h. Primary antibodies were added, and the slides were incubated overnight at 4 °C in a moist chamber. The next morning, the slides were washed three times with 1× PBS (5 min each) and a fluorophore-conjugated secondary antibody was added. The slides were then incubated in the dark for one hour at room temperature in a moist chamber. After washing the slides twice with distilled water, coverslips were applied with Vectashield DAPI mounting media (Vector, H-1200). A list of primary and secondary antibodies that were used for immunofluorescent staining is provided in Supplementary Table S2.

### Cell cultures

Fibroblasts cultures (MEF) were established from wildtype and mutant embryos (E12.5) that carried one or two *Jak1* conditional knockout alleles (*Jak1*^*wt/wt*^. *Jak1*^*fl/wt*^, *Jak1*^*fl/fl*^). The cells were maintained in Dulbecco’s modified Eagle’s medium (DMEM) medium supplemented with 10% fetal bovine serum (FBS), 2 mM glutamine, 0.1 mM nonessential amino acids, 10 mg/ml gentamycin, 100 U/ml penicillin, and 100 mg/ml streptomycin. MEFs were immortalized by serial passaging (3T3 protocol). *Jak1*^*fl/wt*^ and *Jak1*^*fl/fl*^ MEFs were infected with a pBabe-Flp retroviral vector and selected with 7 µg/ml puromycin to establish cell lines that carried *Jak1* conditional knockout alleles with a deletion of the neomycin selection cassette (*Jak1*^*fl-neo/wt*^, *Jak1 *^*fl-neo/fl-neo*^). DNA from cell lines with the *Jak1*^*wt/wt*^. *Jak1*^*fl/fl*^, *Jak1*^*fl-neo/wt*^, and *Jak1 *^*fl-neo/fl-neo*^ alleles were used as positive controls for PCR-based assays to validate the tissue-specific expression and activity of the Flp recombinase in MMTV-Flp *Jak1*^*fl/fl*^ females (Fig. 1).

Mammary tumors from MMTV-Flp *FSF-Kras*^*G12D*^* Rosa26*^*CAG-FSF-CreERT2*^* Jak1*^*fl/fl*^ females were enzymatically dissociated, and isolated cancer cells were cultured in Dulbecco-modified Eagle medium (DMEM/F12) supplemented with insulin, epidermal growth factor, fetal bovine serum, penicillin/streptomycin, and gentamicin. To conditionally delete JAK1, cells were treated for 48 h with 1 µM 4-hydroxytamoxifen (Sigma H6278-10MG). For DNA and immunoblot analyses, the cancer cells were washed with 1× PBS and collected using 0.05% Trypsin–EDTA or by scraping cells in ice-cold 1× PBS.

### Immunoblot analysis

Cell pellets were resuspended in lysis buffer containing 1% Nonidet P-40, 0.5% sodium deoxycholate, 0.1% SDS, 1 mM phenylmethylsulfonyl fluoride, 0.4 units/ml aprotinin, 1 mM NaF, leupeptin, and 0.1 mM sodium orthovanadate, sonicated for 3 s, and placed on ice for 30 min. The extracts were resolved by SDS-PAGE and blotted onto polyvinylidene fluoride (PVDF) membranes (Invitrogen). The membranes were blocked for 1 h at room temperature in 5% Bovine Serum Albumin (BSA) in 1× TBST. Next, the membranes were incubated at 4 °C overnight with primary antibodies in blocking buffer. The membranes were washed three times for 5 min in 1× TBST and incubated for 1 h at room temperature with horseradish peroxidase-conjugated secondary antibodies [goat anti-rabbit (HAF008) from R&D Systems or Digital anti-Mouse-HRP (R1005) from KwikQuant] in blocking buffer. Membranes were washed five times for 5 min with 1× TBST and then two times for 5 min in 1× TBS (Tris-buffered saline without Tween 20) and for 5 min in ultrapure water (Invitrogen 10977-023). Protein bands were detected using the ECL chemiluminescence kit for Western blot analysis [KwikQuant Ultra Digital-ECLTM Substrate Solution (Cat #: R1002)] according to the instructions by the manufacturer (Kindle Biosciences, LCC). Membranes were stripped using a mild glycine stripping buffer (Abcam protocol) for consecutive detection of various proteins. A list of the primary and secondary antibodies that were used for immunoblotting is provided in Supplementary Table S3.

## Supplementary Information

Supplementary Information.
