# Supplementary material for: Dual recombinase action in the normal and neoplastic mammary gland epithelium
Source: Sci Rep. 2021 Oct 21;11:20775. doi: 10.1038/s41598-021-00231-8 (PMC8531329; doi:10.1038/s41598-021-00231-8)
Supplement: Supplementary file 1 — Supplementary Information. [file 41598_2021_231_MOESM1_ESM.pdf]

Supplementary Table 1: PCR primer sets to genotype the various transgenes and genetically engineered alleles as well as to determine Flp and Cre-mediated recombination events.

**Transgene / Allele**	**Primer #**	**Sequence (5' - 3')**	**bp**
MMTV-Flp	2546	CCT GGA ACG GCA TCA TCA GC	275
	2128	CTC CCA TTC ATC AGT TCC ATA GG	
	2127	AGT GAT AGA GCT CTT GCC TAG C	570
	2525	CGT TGT AAG GGA TGA TGG TGA ACT	
Jak1$^{fl/wt}$	2614	CCA TCA GCA CTA GCT GAG GTT C	285 (fl-neo)
(Flp recombined)	2615	CAC AAT GTA GCA AGA CCA AGC CAT G	2,000 (fl)<br>200 (wt)
Jak1$^{fl(-neo)nulll}$	2717	GTA ACT AGC AGA AGG TCT GAT CTG	>900 (fl-neo)
(Flp+Cre recombined, null)	2615	CAC AAT GTA GCA AGA CCA AGC CAT G	220 (null)
CAG-LSL-GFP	2004	GGC TCT AGA GCC TCT GCT AAC C	~270
	2211	GCC ATT GGG ATA TAT CAA CGG TG	
MMTV-neu	1259	TTT CCT GCA GCA GCC TAC GC	590
	1260	CGG AAC CCA CAT CAG GCC	
Rosa26$^{CAG-FSF-GFP}$	2729	CCC AAA GTC GCT CTG AGT TGT TAT C	550 (wt)
	2730	GAA GGA GCG GGA GAA ATG GAT ATG	350 (mu)
	2731	CCA GGC GGG CCA TTT ACC GTA AG	
FSF-Kras^{G12D}	2528	CAC CAG CTT CGG CTT CCT ATT	270 (wt)
	2529	AGC TAA TGG CTC TCA AAG GAA TGT A	350 (mu)
	2530	GCG AAG AGT TTG TCC TCA ACC	
Kras^{G12D}	2600	GTC TTT CCC CAG CAC AGT GCA G	310 (wt)
(Flp recombined)	2602	GTT TTG TAG CAG CTA ATG GCT CTC	344 (mu)
Trp53^{R172H}	2598	AGC CTG CCT AGC TTC CTC AGG	290 (wt)
	2599	CTT GGA GAC ATA GCC ACA CTG	330 (mu)
Rosa26$^{CAG-FSF-CreERT2}$	2534	GAA TGT GCC TGG CTA GAG ATC	190
	2535	GCA GAT TCA TCA TGC GGA	
Rosa26$^{CAG-FSF-CreERT2}$	2729	CCC AAA GTC GCT CTG AGT TGT TAT C	550 (wt)
	2730	GAA GGA GCG GGA GAA ATG GAT ATG	~420 (mu)
	2731	CCA GGC GGG CCA TTT ACC GTA AG	
Rosa26$^{CAG-CreERT2}$	2723	GTT CGG CTT CTG GCG TGT	490
(Flp recombined)	2724	CGA TCC CTG AAC ATG TCC ATC	

Supplementary Table 2: Primary and secondary antibodies for immunofluorescent staining.

**Primary Antibodies**	**SOURCE**	**IDENTIFIER**	**DILUTION**
α-GFP	Avēs Labs	GFP-1020	1:1000
α-CK8	Developmental Studies Hybridoma Bank	TROMAI	1:100
α-CK14	Covance	PRB-155P	1:1000
α-E-cadherin	Cell Signaling	#3195	1:200
α-N-cadherin	Cell Signaling	#14215	1:200
pSTAT3	Cell Signaling	#9145S	1:100
**Secondary Antibodies**	**SOURCE**	**IDENTIFIER**	**Dilution**
Alexa Fluor 488 goat anti-chicken	Invitrogen	A11039	1:1000
Alexa Fluor 488 donkey anti-rat	Invitrogen	A21208	1:1000
Alexa Fluor 594 goat anti-rat	Invitrogen	A11007	1:1000
Alexa Fluor 594 goat anti-rabbit	Invitrogen	A11012	1:1000
Alexa Fluor 594 donkey anti-rabbit	Invitrogen	A21207	1:200-1000
Alexa Fluor 488 donkey anti-rabbit	Invitrogen	A21206	1:1000
Alexa Fluor 594 goat anti-mouse	Invitrogen	A11005	1:1000

Supplementary Table 3: Primary and secondary antibodies for immunoblotting.

**Primary Antibodies**	**SOURCE**	**IDENTIFIER**	**DILUTION**
α-JAK1	Cell Signaling	#50996	1:1000
α-pSTAT3	Cell Signaling	#9145S	1:1000
α-STAT3	Cell Signaling	#9139	1:1000
α-GAPDH	Cell Signaling	#5174S	1:2000
**Secondary Antibodies**	**SOURCE**	**IDENTIFIER**	**DILUTION**
HRP-conjugated goat anti-rabbit	R&D Systems	HAF008	1:2000
Digital anti-Mouse-HRP	KwikQuant	R1005	1:2000

Raw Images of PCR gels or Immunoblots

Figure 1C

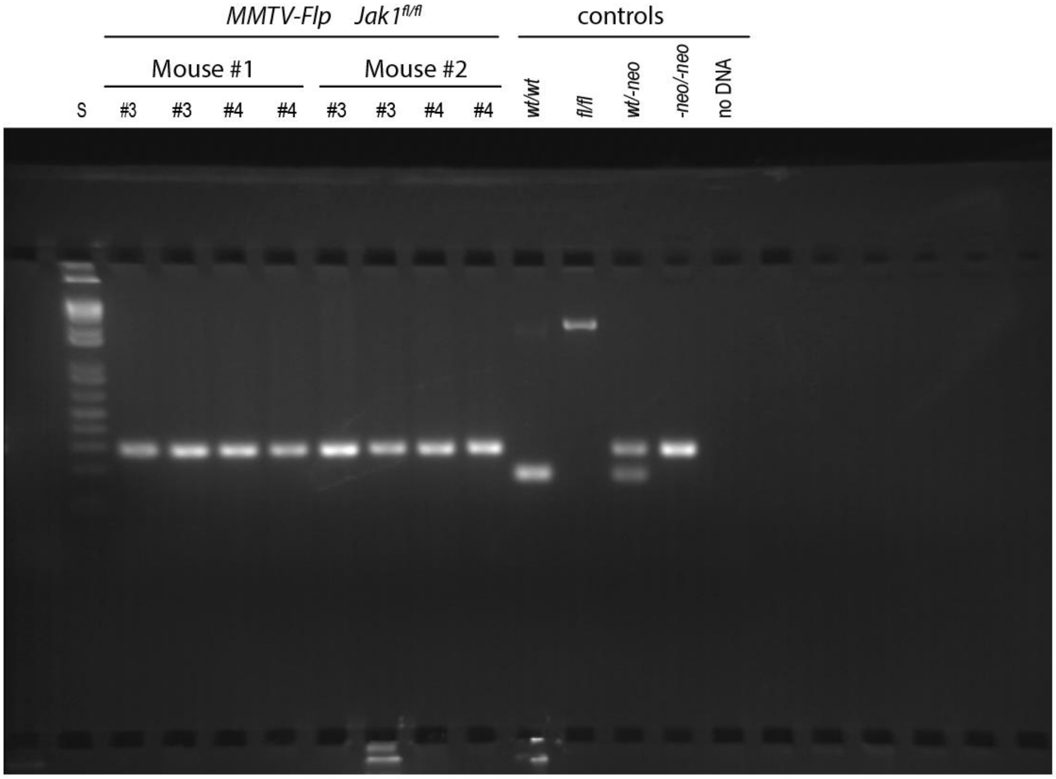

Figure 1D

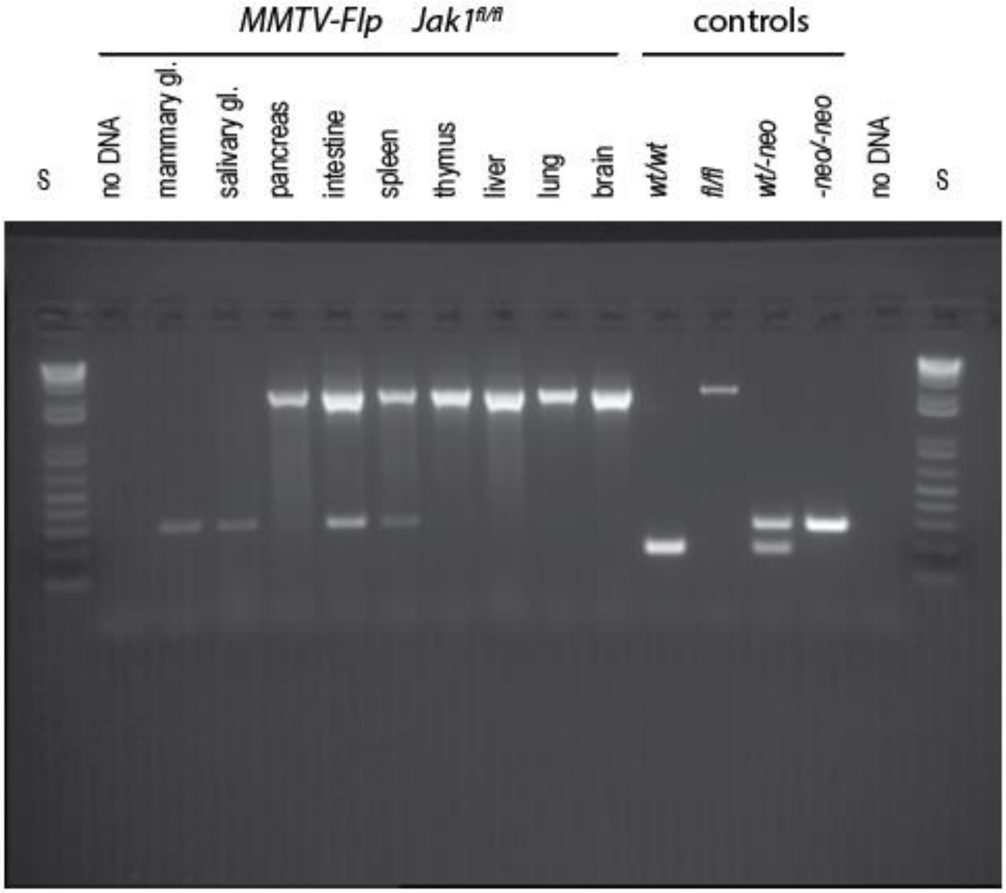

Figure 7A

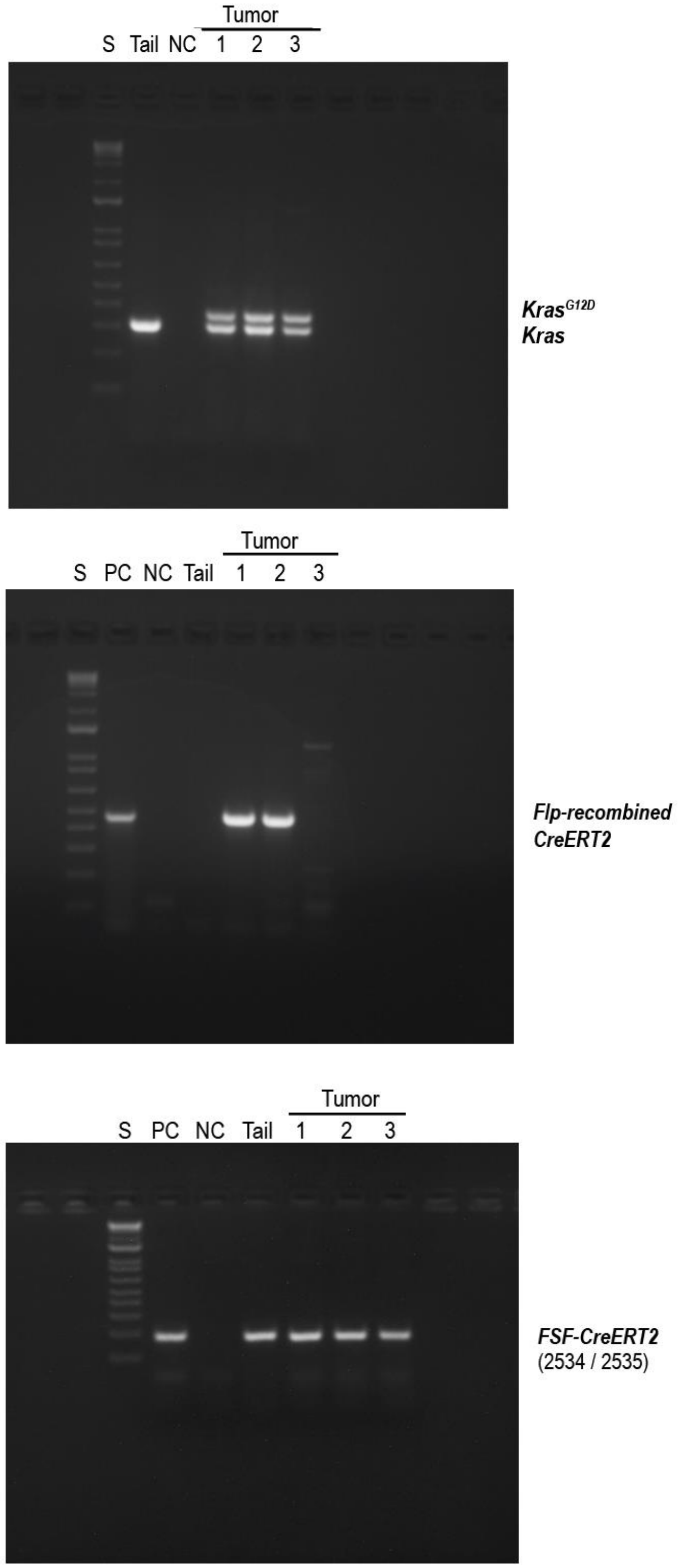
Tumor
S Tail NC 1 2 3
Kras^{G12D}
Kras
Tumor
S PC NC Tail 1 2 3
Flp-recombined
CreERT2
Tumor
S PC NC Tail 1 2 3
FSF-CreERT2
(2534 / 2535)

Figure 7B

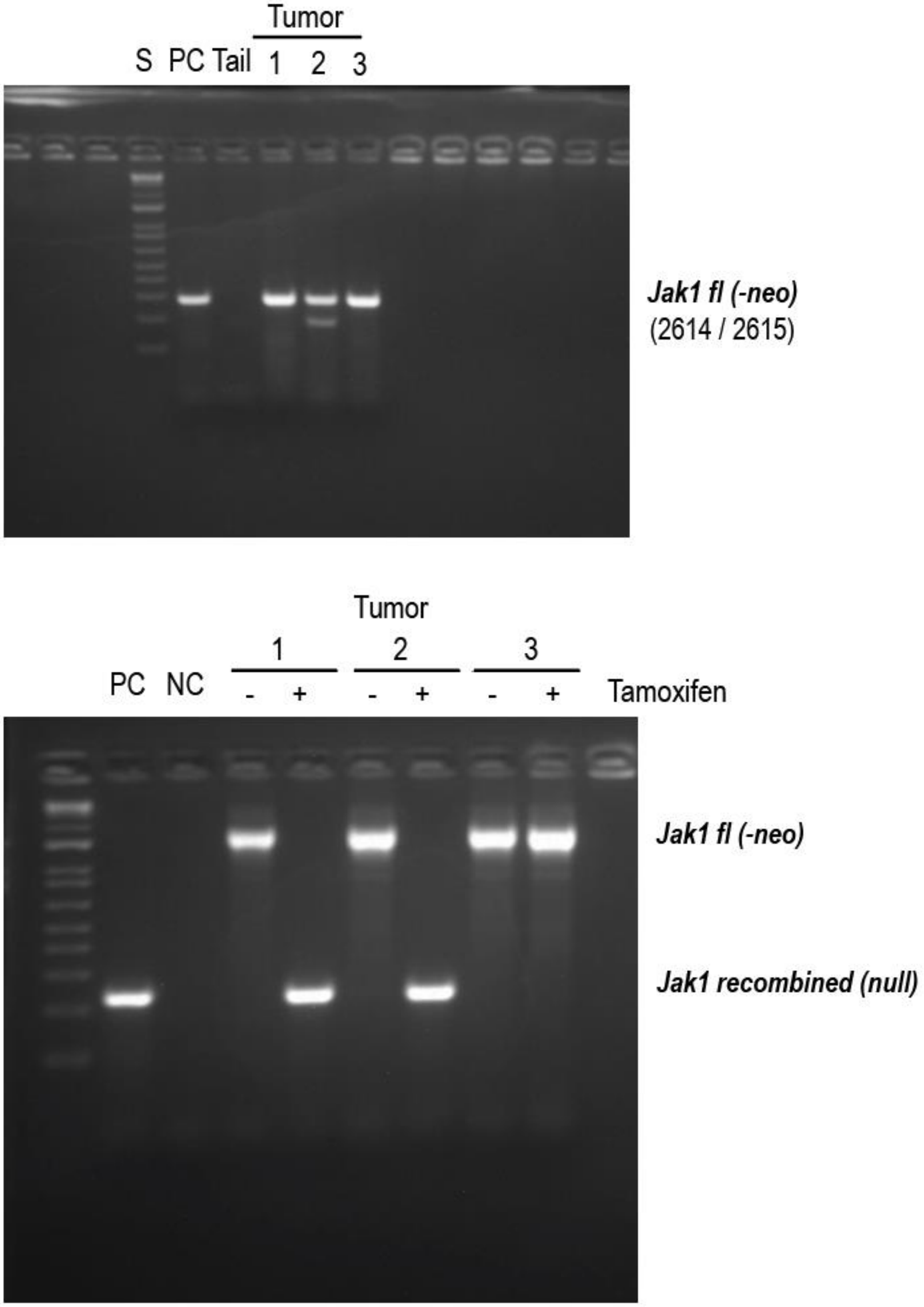

Tumor
S
PC
Tail
1
2
3
Jak1 fl (-neo)
(2614 / 2615)
Tumor
1
2
3
PC
NC
-
+
-
+
-
+
Tamoxifen
Jak1 fl (-neo)
Jak1 recombined (null)

Figure 7C

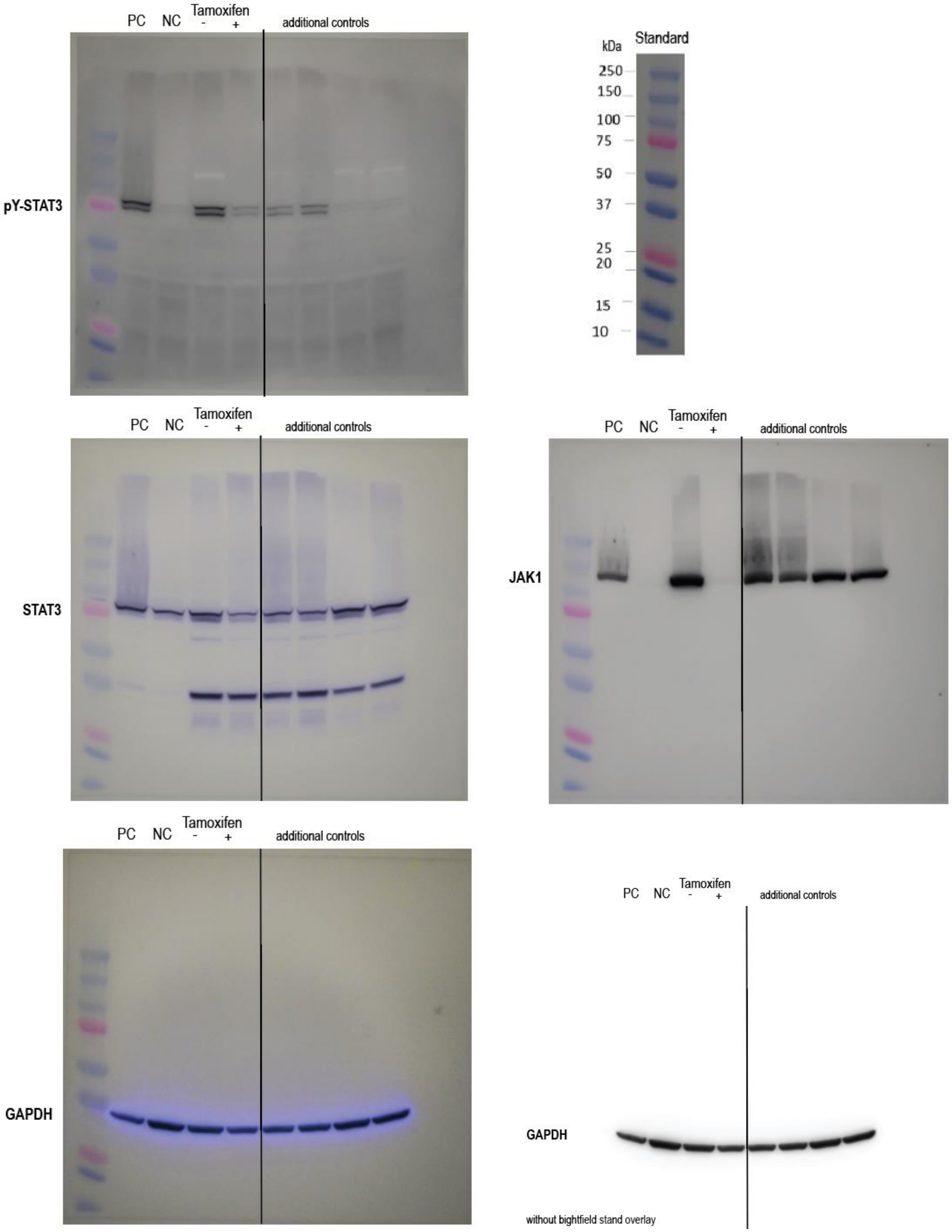
PC
NC
Tamoxifen
-
+
additional controls
pY-STAT3
kDa
Standard
250
150
100
75
50
37
25
20
15
10
STAT3
JAK1
GAPDH
without bightfield stand overlay
